# Mindfulness-based interventions to reduce anxiety among Chinese college students: A systematic review and meta-analysis

**DOI:** 10.3389/fpsyg.2022.1031398

**Published:** 2023-01-06

**Authors:** Jun Li, Can Xu, Keyan Wan, Yihong Liu, Liu Liu

**Affiliations:** ^1^School of Social and Behavioral Sciences, Nanjing University, Nanjing, China; ^2^Monetware Inc., Shanghai, China; ^3^Department of Sociology, School of Public Affairs, Nanjing University of Science and Technology, Nanjing, China

**Keywords:** mindfulness-based interventions, anxiety, Chinese college students, meta-analysis, systematic review

## Abstract

Mindfulness-based interventions are found to have a positive effect on an individual’s mental health. Using a meta-analysis method, this study examined the effects of mindfulness-based interventions on alleviating anxiety among Chinese college students. Using six international and two Chinese electronic databases, we comprehensively researched literature published between 1 January 2012 and 31 December 2021. The literature was then carefully selected and classified. The literature selection, data extraction, and quality assessment were all independently conducted by two members of the research team; any disagreements were resolved through consultation with a third researcher. A total of 11 original articles met all the eligibility criteria and were included in the meta-analysis. The meta-analysis revealed that mindfulness-based interventions have a positive effect on the remission of anxiety among Chinese college students. This confirms the need to generalize mindfulness-based interventions as a form of treatment and prevention for anxiety among Chinese college students.

## 1. Introduction

Anxiety is an unpleasant emotional state which involves the feeling of fear, tension, entanglement, and irritability (Yi et al., 2022). Anxiety is often studied alongside depression, which is another psychiatric condition. Together, they are the most common psychiatric conditions in a general medical setting (Carek et al., 2011). There are a wide range of studies that focus on the interventions used to alleviate depression, or the combination of depression and anxiety (Hofmann and Gómez, 2017). However, few studies focus solely on anxiety hence why this study is unique. Slightly different from depression (Renner et al., 2018), anxiety is caused by the excessive worry of a potentially miserable situation (Pang et al., 2019), which can include lower-income, work impairment, decreased quality of life and negative effects on family relationships (Roemer et al., 2013).

Anxiety disorder has already become a serious public health problem in China. Young people who enter higher education are particularly affected (Cheng et al., 2020). Indeed, one third of 6,032 Chinese college students were found to have mild to very severe anxiety (Yu et al., 2022). Although a few experiences of anxiety are found to have a positive impact on an individual (Yi et al., 2022), chronic anxiety can lead to negative mental and physical health status, which can further affect a student’s studies and future career development (Zhang et al., 2021; Yu et al., 2022). Hence, there is a need for effective interventions to treat anxiety-related physical and psychological problems faced by college students in China (Li and Qin, 2021). This study plans to focus on anxiety among Chinese college student due to the awareness of the following three factors: (1) high prevalence of anxiety among Chinese college students; (2) current low levels of intervention within Chinese universities, and amongst families and society as a whole; (3) low levels of attention to the phenomenon of anxiety among Chinese university students when compared to other Western societies.

Multiple interventions can be used to relieve anxiety. An effective psychotherapy treatment is mindfulness-based intervention; it is also found to help other mental health problems (Marchand, 2012; Liu Y.-C. et al., 2021). Mindfulness originally developed from Buddhist traditions that combine contemplation and meditation (Intarakamhang et al., 2020), and is even defined by some as a state of consciousness (Grossman et al., 2015). Practicing mindfulness entails experiencing the present moment, rather than trying to change anything (Marchand, 2012; Hofmann and Gómez, 2017; Sun et al., 2021). This present-centered self-awareness skill can be further developed through regular exercises (Repo et al., 2022), eventually enabling the individual to release certain frustration-like habitual reactions (Liu X. et al., 2021) such as anxiety. Mindfulness could also be considered as the composition of both self-regulation of attention and an orientation to the present moment, characterized by curiosity, receptivity, and openness (Hofmann and Gómez, 2017). It is confirmed that mindfulness is effective at improving emotional wellbeing (Zhu et al., 2019). In mindfulness-based interventions, instructors help individuals increase their awareness and tolerance of thoughts and emotions through the provision of training, including guided routines, which help individuals to develop healthy and habitual problem-focused coping mechanisms (Lo et al., 2022).

Mindfulness-based interventions emphasize a non-judgmental focus on and awareness of the present moment, self-regulation of emotions and behavior, and mindfulness meditation practice (Crane et al., 2017). Currently, the most developed and commonly used mindfulness-based interventions include mindfulness-based stress reduction therapy (MBSR) (Keng et al., 2020), mindfulness-based cognitive therapy (MBCT) (Compen et al., 2018), acceptance and commitment therapy (ACT) (McCracken and Vowles, 2014), and dialectical behavior therapy (DBT) (Wang and Jiang, 2016). First-generation mindfulness-based interventions such as MBSR and MBCT focus on both mindfulness awareness and mindfulness meditation training (Kocovski et al., 2009). However, later interventions such as ACT and DBT only emphasize mindfulness consciousness and ignore a formal mindfulness meditation practice (Hayes et al., 1999; Gilbert, 2018). The last few years have seen an increasing interest in the efficacy of mindfulness-based interventions (Khoury et al., 2013); evidence suggests they can be effective in adults with a range of mental and medical conditions including anxiety disorders (Evans et al., 2008; Vøllestad et al., 2012; Borquist-Conlon et al., 2017). For example, a recent meta-analysis has investigated the effect of mindfulness-based intervention on anxiety disorders, reporting a moderate and significant effect on reducing anxiety symptoms (Borquist-Conlon et al., 2017). In addition, initial research has found that mindfulness-based intervention offers a feasible and promising form of intervention for youths and suggest a possibility of the focus of youth in clinical setting for the future research (Burke, 2010; Zoogman et al., 2015).

Current anxiety intervention programs regarding Chinese college students comprise two main categories: physical activities and psychotherapeutic interventions. Physical activities generally include *Taijiquan*, *Baduanjin*, Five-Animal Play, and other traditional Chinese medical health practices (Zhang et al., 2021). Psychotherapeutic interventions include motivational interviewing, self-compassion intervention, forgiveness intervention, *Zhong-Yong* thinking-based DBT, and cognitive-behavioral intervention (Zhang et al., 2014; Cui et al., 2016; Li and Ren, 2019; Pan et al., 2020; Yang X. et al., 2020; Huang et al., 2021). Although there is an existing meta-analysis on the effects of physical activity interventions on alleviating anxiety among Chinese college students (Zhang et al., 2021), scarce meta-analysis evidence has identified any effective psychotherapeutic intervention.

Although a few existing empirical studies focus on the effectiveness of mindfulness-based interventions in the Chinese context (Gu et al., 2016; Sun et al., 2021), little attention has been given to synthesizing the evidence on outcomes of mindfulness-based interventions on reducing anxiety, especially among college students (Lei et al., 2022). In view of the research gap in existing literature, this study conducts a meta-analysis that provides a systematic review of previous studies, and synthesizes the evidences to show whether mindfulness-based interventions are useful in alleviating anxiety among Chinese college students, compared to those with no or other types of interventions. This study has two aims: (1) provision of a reliable empirical basis for the development of interventions to reduce anxiety disorder among Chinese college students; (2) establishment of effective clinical evidence for the application of mindfulness-based interventions for researchers and educators in China. Ultimately, this study provides a comprehensive and transparent examination of existing literatures in order to identify gaps in the current evidence-based research, which can then serve as a guide for future research and practice.

Specifically, this study aims to research the combination of mindfulness with meditation with a particular focus on MBSR and MBCT. MBSR was developed in the late 1970s and places emphasis on stress reduction and improvements in wellbeing; it has been applied widely across physical health, mental health and non-clinical populations (Strauss et al., 2014). MBCT was developed in the 1990s and has been extended to people with current diagnoses of anxiety disorders in recent years.

## 2. Materials and methods

The present meta-analysis is reported according to the guidelines in the Preferred Reporting Items for Systematic Review and Meta-analyses (PRISMA) 2020 statement (Page et al., 2021). The randomized controlled trial (RCT) studies included in this review are all published articles, hence informed consent and ethical approval were unnecessary.

### 2.1. Search strategy

The comprehensive literature search was conducted using six international and two Chinese electronic databases, including Web of Science (WOS), PubMed, EMBASE, PsyCINFO, Scopus, Cochrane Library, Wanfang Data, and China National Knowledge Infrastructure (CNKI). Only articles published between 1 January 2012 and 31 December 2021 were eligible for consideration because studies on mindfulness-based interventions for anxiety among Chinese college students have increased over the past decade. The search terms consisted of three subsets: participant (“college students” and “university students”); intervention (“mindfulness” and “meditation” and “MBSR” and “MBCT” and “mindfulness-based intervention” and “mindfulness-based stress reduction” and “mindfulness-based cognitive therapy”); country restriction (“China” and “Chinese”). We combined the terms with two Boolean operators (i.e., AND and OR) to search for relevant articles in both English and Chinese. The final search string is shown in Table 1.

**TABLE 1:** Final search string.

	Research terms
Participant	“College students” and “university students”
Intervention	“Mindfulness” and “meditation” and “mindfulness-based stress reduction therapy (MBSR)” and “mindfulness-based cognitive therapy (MBCT)” and “mindfulness-based intervention” and “mindfulness-based stress reduction” and “mindfulness-based cognitive therapy”
Country	“China” and “Chinese”

### 2.2. Inclusion and exclusion criteria

Articles that met the following four criteria were included in this systematic review: (1) the study design had to be RCT; (2) the experimental sample had to be Chinese college students; (3) the intervention strategy used in the experimental group had to be mindfulness-based intervention methods that included meditation, MBSR, or MBCT; (4) the primary outcome had to be anxiety, but with no specific measurement.

Articles were then examined based on the following exclusion criteria: (1) articles that were neither Chinese nor English; (2) repeated articles; (3) articles without full text available; (4) articles that did not report anxiety as the outcome; and (5) articles with control groups that use a mindfulness-based intervention.

### 2.3. Study selection

The literature screening and selection were conducted independently by two of the researchers in accordance with the PRISMA statement (Page et al., 2021). They first screened the titles and abstracts of the candidate articles and excluded irrelevant articles. Second, they fully read the remaining texts in order to choose which articles met the inclusion criteria of this meta-analysis. Any disagreements between the two researchers were addressed through consultation with a third researcher. EndNote X20 software was used for the selection and classification of articles.

### 2.4. Date extractions

Following the selection of eligible studies, the two researchers independently extracted data in accordance with the format designed by Sturt et al. (2012). A data extraction form was then generated to summarize the basic characteristics of the included studies, such as the literatures (e.g., title, author, abstract, and year of publication), the participants (e.g., age and gender), the intervention and control groups (e.g., method of intervention, number of interventions, and the characteristics of control groups), and the outcome measures [e.g., anxiety scale, mean, and standard deviation (SD)]. A third researcher intervened whenever any differences existed between the two researchers.

### 2.5. Quality assessment

The two researchers independently evaluated the literature quality by using the Cochrane risk of bias (RoB) assessment tool (Higgins et al., 2019). Any discrepancies in the assessment were discussed by the two researchers, and a third researcher participated to re-check evaluation results and help reach a consensus. A total of six domains of RoB were evaluated in the following contents: (1) selection bias (e.g., sequence generation and allocation concealment); (2) performance bias (blinding of participants and personnel); (3) detection bias (blinding of outcome assessors); (4) attrition bias (incomplete outcome data); (5) reporting bias (selective outcome reporting); and (6) other (other potential threats to validity) (Higgins et al., 2019). The RoB for each domain was assessed as low, high, or unclear. Studies with a high RoB were considered to be of low quality.

### 2.6. Data analysis

Review Manager 5.4 software was used to perform the meta-analysis of the effectiveness of mindfulness-based interventions. We selected the data (mean and SD) from all articles that provided pre-to-post intervention scores. *I*^2^ was used to examine the heterogeneity across literature included in quantitative statistics.

When *I*^2^ ≥ 50%, *p* < 0.10, i.e., there is high heterogeneity among eligible literatures, hence a random-effect model is chosen to report the results; the fixed-effect model is adopted when *I*^2^ < 50% (Chen et al., 2021). When using a fixed-effect model, it is assumed that the population effect sizes are the same across all studies (Cheung et al., 2012). Contrastingly, the random-effect model attempts to generalize findings beyond the included studies by assuming that the selected studies are random samples from a larger population (Lim et al., 2018).

Accordingly, the influence of heterogeneity led us to utilize a random-effect model to carry out this meta-analysis. Subgroup analysis was used to explore the sources of heterogeneity. In addition, a high heterogeneity (*I*^2^ ≥ 50%, *p* < 0.10) usually requires a subgroup analysis and sensitivity analysis (Shou et al., 2022). Therefore, a subgroup analysis was used to explore the sources of heterogeneity, and a sensitivity analysis was used to “investigate the influence of a single study on the overall pool estimation” (Bai et al., 2022). With regards to different anxiety assessment methods, standard mean differences (SMDs) with 95% confidence intervals (CIs) are applied to analyze the levels of anxiety among Chinese college students. A bilateral *p* < 0.05 is considered statistically significant in the overall effect. The conducted sensitivity analysis is used to report the stability of outcomes. In analyses that included 10 studies or more, we generated the funnel plot and Egger’s test to assess the publication bias.

## 3. Results

### 3.1. Study selection outcomes

**FIGURE 1:**
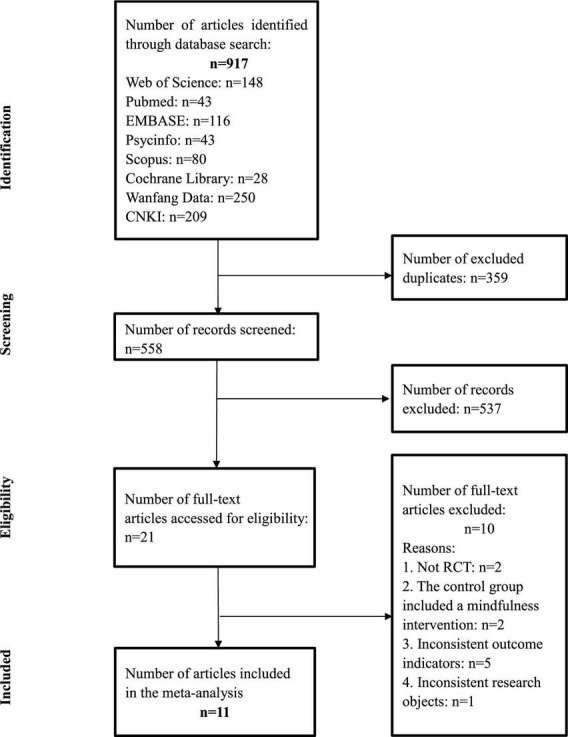
PRISMA diagram of included studies in the meta-analysis.

Initially, 917 articles were identified from six English and two Chinese databases, of which 359 duplicates were removed and the remaining 558 articles were exported to EndNote X20 software for further screening and selection. The abstracts and titles were screened and 537 articles were then excluded based on the inclusion and exclusion criteria. After a full text review of the remaining 21 articles, a further 10 studies were excluded for the following reasons: (1) the study design was not RCT (*n* = 2); (2) the control group included a mindfulness-based intervention (*n* = 2); (3) the study revealed inconsistent outcome indicators (*n* = 5); and (4) the study had inconsistent research objects (*n* = 1). Eventually, 11 articles were included in this review. A PRISMA flow diagram was produced to record the literature selection process (see Figure 1).

### 3.2. Risk of bias

**FIGURE 2:**
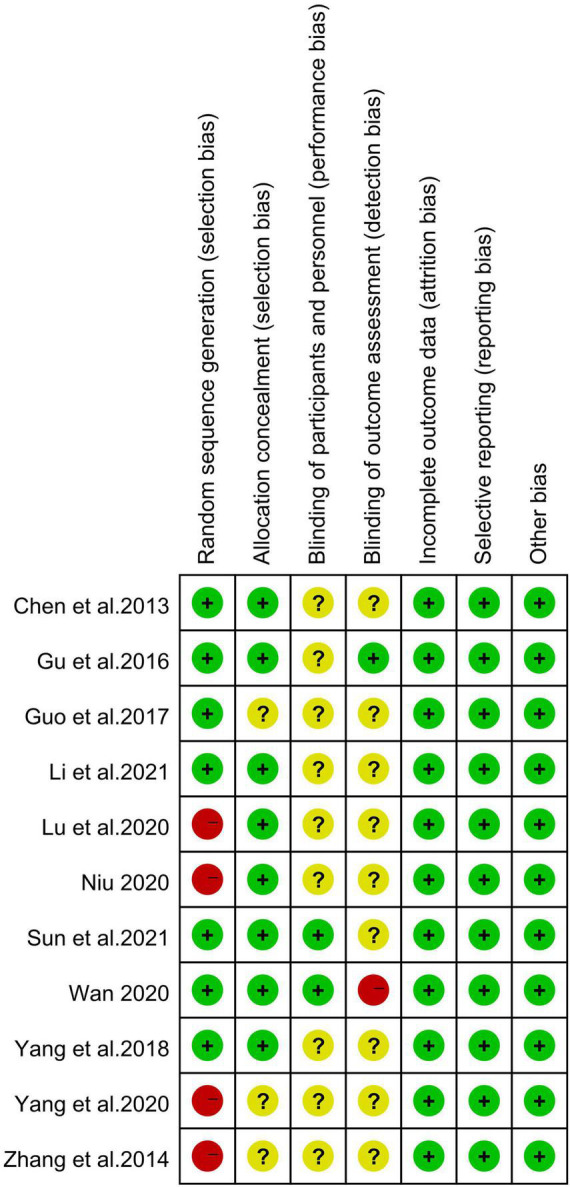
Literature quality evaluation using Cochrane risk of bias (RoB) assessment tool.

Figure 2 shows a summary of the RoB for the included studies. The 11 studies were all evaluated as having a low or medium RoB. A random sequence generation saw four of the studies evaluated as having a high RoB. Three studies did not report the status of allocation concealment (e.g., Guo and Wang, 2017). Nine studies did not report the blinding of participants and personnel (e.g., Chen et al., 2013). One study was rated with a high risk of selection bias based on the blinding of outcome assessment (Wan, 2020), and nine were unclear (e.g., Chen et al., 2013). All studies were considered to have a low RoB in terms of random sequence generation, measurement results, data reporting, and other bias.

### 3.3. Characteristics of studies

Table 2 summarizes the basic characteristics of the 11 systematically reviewed RCT studies. Based on the inclusion and exclusion criteria, all of the included studies were published between 2013 and 2021, and were conducted in China. Study sample sizes range between 54 (Gu et al., 2016) and 800 (Niu, 2020). The total sample size is 1,602, with 801 participants in the experimental groups, and the remaining 801 in the control groups. Seven different types of stress scales were used in the 11 studies to evaluate the outcome of anxiety: (1) Self-rating Anxiety Scale (SAS) (used in five studies, e.g., Chen et al., 2013); (2) Hamilton Anxiety Scale (HAMA) (Wan, 2020); (3) Depression Anxiety Stress Scale-21 (DASS-21) (Yang L. et al., 2020); (4) Symptom Checklist-90 (SCL-90) Scale (Lu et al., 2020); (5) Beck Anxiety Inventory (BAI) Scale (Gu et al., 2016); (6) Affective Control Scale (ACS) (Li and Qin, 2021); and (7) General Anxiety Disorder-7 (GAD-7) Scale (Sun et al., 2021).

**TABLE 2:** Basic characteristics of the included studies.

References	Participants	Country	Number of participants (E/C)	Mean age	Treatment type		Duration of intervention	Outcome measure
					Control group	Experimental group		
Wan, 2020	Class of 2018 students at a university	China	30/30	19.57	No intervention	Mindfulness group intervention	2 weeks	Hamilton Anxiety Scale (HAMA)
Yang L. et al., 2020	College students during COVID-19	China	53/51	18.6	No intervention	Mindfulness-based stress reduction therapy (MBSR)	10 days	Depression Anxiety Stress Scale-21 (DASS-21)
Lu et al., 2020	Medical undergraduates	China	40/40	19.78	No intervention	Internet-based, self-management cognitive behavior therapy	8 weeks	Symptom Checklist-90 (SCL-90)
Niu, 2020	College students with cell phone dependence	China	400/400	22.135	Usual care	Mindfulness group intervention	2 months	Self-rating Anxiety Scale (SAS)
Zhang et al., 2014	College students with cell phone dependence	China	30/30	22.02	No intervention	Mindfulness-based cognitive therapy (MBCT)	4 weeks	SAS
Yang et al., 2018	College students with cell phone dependence	China	60/56	–	Usual care	MBSR	8 weeks	SAS
Guo and Wang, 2017	Freshmen	China	28/30	–	No intervention	Mindfulness meditation therapy	8 weeks	SAS
Gu et al., 2016	Undergraduate students	China	28/26	20.29	Delayed intervention	MBCT	6 weeks	Beck Anxiety Inventory (BAI)
Sun et al., 2021	Undergraduate students	China	52/52	22.21	Delayed intervention	An intervention combining mindfulness-based stress reduction and mindfulness-based cognitive	1 month	General Anxiety Disorder-7 (GAD-7)
Chen et al., 2013	Chinese nursing students	China	30/30	19.5	Social support-based intervention	Mindfulness meditation therapy	1 week	SAS
Li and Qin, 2021	Undergraduate students	China	50/56	21.04	No intervention	MBSR	6 weeks	Affective Control Scale (ACS)

#### 3.3.1. Characteristics of participants

The participants in the included studies were all college students in China. The mean age of the sample participants in the nine studies ranged from 18.6 to 22.21 years; the other two studies did not report the age of participants (Guo and Wang, 2017; Yang et al., 2018). Two of the studies questioned participants during the COVID-19 pandemic (Yang L. et al., 2020; Sun et al., 2021). In three studies, the participants were medical and nursing undergraduates (Chen et al., 2013; Yang et al., 2018; Lu et al., 2020). In two studies, participants were identified as college students with anxiety problems caused by cell phone over-use (Zhang and Zhu, 2014; Niu, 2020). These students were proven to have a higher level of anxiety when not using their cell phone.

#### 3.3.2. Characteristics of intervention and control groups

The degree of mindfulness-based intervention duration varied across the 11 studies, ranging from 10 days (Yang L. et al., 2020) to 8 weeks (Guo and Wang, 2017; Yang et al., 2018; Lu et al., 2020; Niu, 2020). Regarding the intervention format, two studies provided participants with mindfulness-based interventions in an online format (Yang L. et al., 2020; Sun et al., 2021), while the nine remaining study interventions were conducted in a face-to-face format. With regards to who implemented the interventions, three studies were implemented by trainers with experience in mindfulness (Chen et al., 2013; Gu et al., 2016; Yang et al., 2018), two were conducted by experienced university professors and students in this area (Lu et al., 2020; Li and Qin, 2021), one was conducted by experienced research assistants trained by the first author (Sun et al., 2021), and the remaining five studies either made no mention of who implemented the interventions or simply stated that the interventions were implemented by the trainers (Zhang and Zhu, 2014; Guo and Wang, 2017; Niu, 2020; Wan, 2020; Yang L. et al., 2020).

There were four types of interventions used in the 11 studies: (1) mindfulness-based stress reduction therapy, i.e., MBSR (Yang et al., 2018; Lu et al., 2020; Yang L. et al., 2020; Li and Qin, 2021); (2) mindfulness-based cognitive therapy, i.e., MBCT (Zhang and Zhu, 2014; Gu et al., 2016); (3) MBSR combined with MBCT (Sun et al., 2021); (4) mindfulness with meditation therapy in general (Chen et al., 2013; Guo and Wang, 2017; Niu, 2020; Wan, 2020).

Regarding the characteristics of the control groups, two studies provided participants with usual care (Yang et al., 2018; Niu, 2020), one study provided social support intervention (Sun et al., 2021), and the other eight studies provided either a delayed intervention or no intervention.

### 3.4. Results of the effects of mindfulness-based intervention

#### 3.4.1. Overall effects

**FIGURE 3:**
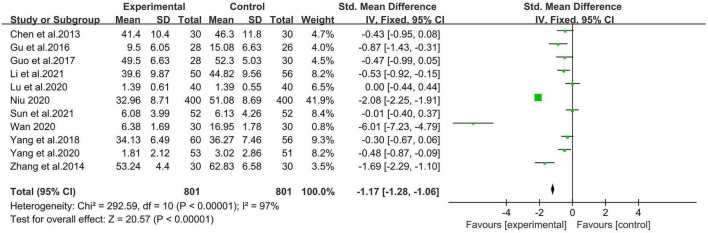
Forest plot of total effects of mindfulness-based intervention on anxiety of Chinese college students.

A meta-analysis of the 11 studies showed that mindfulness-based interventions overall alleviated anxiety among Chinese college students. Figure 3 shows that mindfulness-based intervention results in a decrease in anxiety scale scores among participants, with a mean decrease of 1.07 points (95% CI −1.73 to −0.42). For the sample of 1,602 participants, the overall combined effect corresponding to mindfulness-based interventions was statistically significant (*p* < 0.05), which demonstrates the effectiveness of mindfulness-based intervention on reducing anxiety in Chinese college students. There was found to be large heterogeneity between studies (Chi^2^ = 292.59, *p* < 0.05, *I*^2^ = 97%).

#### 3.4.2. Results of subgroup analysis

**FIGURE 4:**
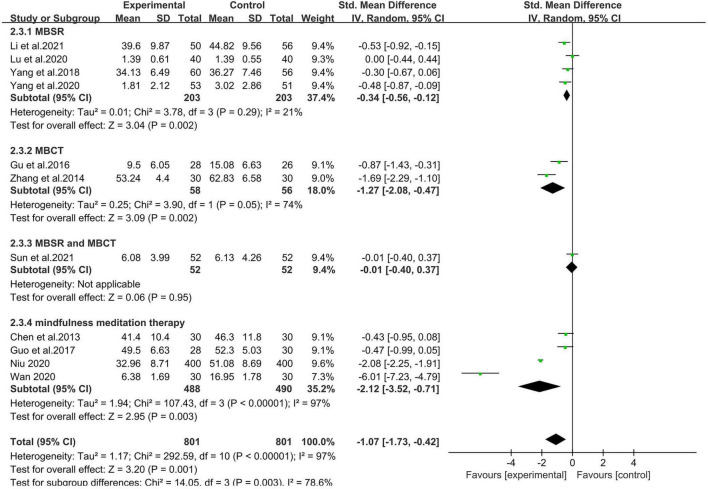
Forest plot of effects of mindfulness intervention based on intervention types.

In the subgroup analysis, we presented the effects of mindfulness-based interventions according to the intervention type. As discussed in the previous section about the intervention method, four studies used MBSR, two used MBCT, one used MBSR and MBCT, and the rest four used mindfulness with meditation therapy in general. As suggested in Figure 4, the subgroups were significantly different (Chi^2^ = 14.05, *p* < 0.05, *I*^2^ = 78.6%), which indicates a statistically significant subgroup effect. The subgroups with intervention methods of MBSR and MBCT were not statistically significant, which moderate unexplained heterogeneity (SMD = −0.01, 95% CI −0.40 to 0.37, *p* > 0.05).

#### 3.4.3. Results of sensitivity analysis

**FIGURE 5:**
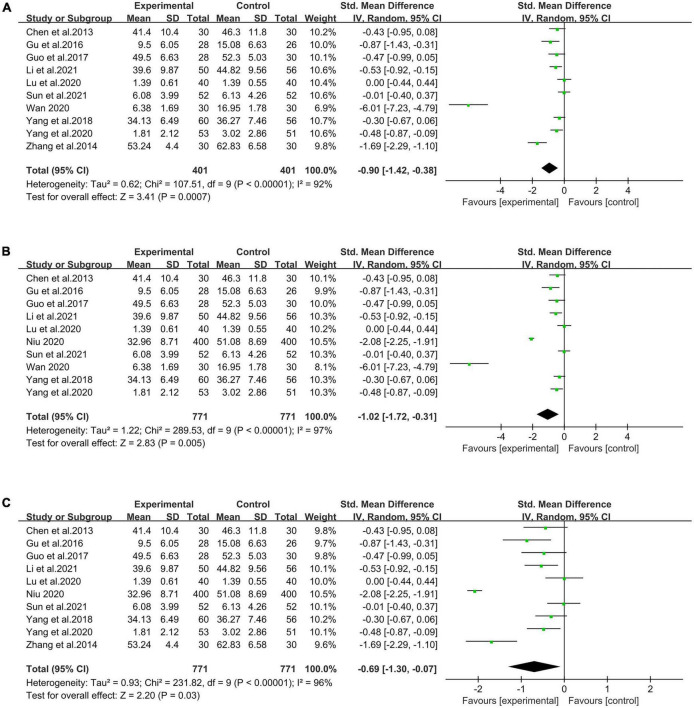
Forest plot of effects of sensitivity analysis. **(A)** Sensitivity analysis without Niu (2020); **(B)** sensitivity analysis without Zhang and Zhu (2014); **(C)** sensitivity analysis without Wan (2020).

In order to test the stability of the results of this meta-analysis, we excluded the study with an obvious larger sample size to test its impact on the overall conclusion, as large differences in sample size between the studies will cause bias (Nakagawa et al., 2021). As shown in Table 2, Niu’s (2020) study included a sample of 800 participants (400 in the experimental group and 400 in the control group), which was noticeably larger than that of the other studies. After excluding this study (see Figure 5A), the overall effect was proven to be significant (*p* < 0.01). The result was consistent with the one before the sensitivity analysis, indicating that the conclusion of this combination was reliable.

Moreover, Zhang and Zhu’s (2014) study was the only one with high risk because it reported on the complexity of participants’ absence. After excluding this study (see Figure 5B), the overall effect was shown to be significant (*p* < 0.01), which was consistent with the results before the sensitivity analysis. It reflected the reliability of the entire conclusion of this combination.

Of the 11 studies, only Wan’s (2020) study reported a high risk of selection bias on the blinding of outcome assessment. After excluding this study (see Figure 5C), the results showed *p* < 0.05, indicating that the overall effect was significant. It was also consistent with the findings before the sensitivity analysis, showing that the overall result of the combination was reliable.

## 4. Discussion

This study used a systematic literature review and meta-analysis to summarize the practical evidence regarding the effectiveness of the mindfulness-based interventions in reducing anxiety among Chinese college students. Specifically, four types of interventions were summarized within the 11 studies. The first type is mindfulness-based stress reduction therapy, i.e., MBSR. This type is proven in reducing fear of emotions in university programs (Li and Qin, 2021); improving mental health and sleep quality (Lu et al., 2020; Yang L. et al., 2020); reducing anxiety level, improving self-control ability and learning effect of medical undergraduates in the military academy (Yang et al., 2018).

The second type is mindfulness-based cognitive therapy, i.e., MBCT. This type is confirmed in improving participants’ ADHD symptoms, mindfulness, and sustained attention (Gu et al., 2016); and reducing the level of college students’ mobile phone over use (Zhang and Zhu, 2014).

The third type is MBSR combined with MBCT. This type is shown promise in reducing distress among young adults in quarantine and addressing the psychological consequences of the pandemic (Sun et al., 2021).

The last type is mindfulness with meditation therapy in general. This type is beneficial for Chinese nursing students in reducing anxiety symptoms and lowering systolic blood pressure (Chen et al., 2013); relieving the anxiety and reducing the loneliness among the college students with mobile phone overuse problem (Niu, 2020); improving the general mental health of college students (Wan, 2020); and with Satya meditation has been found to be useful as a mindfulness training model to effectively intervene in the mental health of college students (Guo and Wang, 2017).

Based on the analysis of the 11 selected articles, mindfulness-based interventions were shown to have an overall significant positive effect on alleviating anxiety levels among Chinese college students. In addition, after excluding the data from Niu (2020) due to a larger sample size, data from Zhang and Zhu (2014) due to the risky-high and the complexity of participants’ absence, and finally, data from Wan (2020) due to the selection bias on blinding of outcome assessment, the results still indicated that the overall effect was significant (*p* < 0.01, *p* < 0.01, *p* < 0.05), which was consistent with the findings foregoing sensitivity analysis, and confirmed the overall stability in the result of the combination. It is noteworthy that anxiety and its mental health implications among college students is a common concern worldwide (Halladay et al., 2018). In line with findings in much western research (Smit and Stavrulaki, 2021), this study concludes that mindfulness-based interventions are indeed beneficial to reducing anxiety among college students.

However, it should be noted that the quality of the literature is unbalanced. Use of the Cochrane RoB assessment tool (Higgins et al., 2019) in the preceding sections revealed different levels of bias within existing studies, such as the RoB in terms of random sequence generation, measurement results, and data reporting. For example, Wan’s (2020) study was rated as high risk of selection bias on blinding of outcome assessment, indicating it was not a high-quality study, which would affect the accuracy of the research results. In this case, this study’s findings should be considered preliminary. More in-depth higher quality empirical research is thus required in the future.

There are also another thing worth considering in future research and practices. The 11 studies use seven stress scales for the assessment of students’ anxiety levels. These scales may have more or less disadvantages when assessing the anxiety levels of Chinese college students. For example, as the SAS scale’s sensitivity and specificity rely on self-assessments rather than medical records, the validity of the self-assessment may cause some bias (Dunstan and Scott, 2020). Moreover, as our target population is Chinese college students, cultural variation may affect the use of the DASS-21 scale due to the lack of validation among Asian samples (Oei et al., 2013). Additionally, the GAD-7 scale has been criticized for only focusing on one type of anxiety disorder and ignoring others such as social phobia (Spitzer et al., 2006). Moreover, the use of multiple measurements is acceptable (McShane and Böckenholt, 2021) but may still cause some bias to the results. The sensitivity and specificity for different stress scales may also be misleading. Furthermore, the complicities of different scales mean this study is not subdivided according to the levels of anxiety disorder. For example if the student were diagnosed with anxiety disorder or to what extend is their anxiety level. Due to the limitation of all existing stress scales, and Chinese students even possibly to develop different stresses within college rather than elsewhere, there is currently no recommended measurement for assessing anxiety among Chinese college students. Therefore, further research is required in order to validate these scales and decrease statistical heterogeneity.

Upon overall reflection, this study has certain limitations due to the constraints of time, knowledge, and academic resources. The primary concern is the heterogeneity. Large heterogeneity was found between the studies (Chi^2^ = 292.59, *p* < 0.05, *I*^2^ = 97%). This may be due to differences in academic competency amongst the researchers and the differences between specific intervention methods. The heterogeneity could influence the results of the meta-analysis with regards to the effectiveness of the mindfulness-based interventions.

Although with limitations, this study still make contributions to not only synthesize the effectiveness of mindfulness-based interventions on Chinese college students’ anxiety alleviation, but also identify the possible research gap that might lead to future’s empirical research. Particularly, since the differences in the educational system within China may contribute to different levels of stress and anxiety, the generalization of these research findings should be carefully considered. The reasons behind anxiety in Chinese college students could be further researched, such as the link with problematic cell phone use. Researchers could also pay attention to (1) potential gender differences in the effectiveness of the mindfulness-based intervention in reducing anxiety; and (2) which mindfulness-based interventions are most effective for different types of college student populations (e.g., students with different majors).

## 5. Conclusion

The results from the meta-analysis and evidence from prior studies demonstrate that mindfulness-based interventions significantly decrease anxiety scale scores among Chinese college students. Mindfulness-based intervention is thus a proven form of psychotherapy that offers effective treatment for anxiety. Higher education policymakers in China should focus more on the mental and physical health of college students and may use this study as a reliable empirical basis for developing interventions for reducing anxiety disorder issues amongst students.

## Author contributions

JL and LL: conceptualization, methodology, supervision, and project administration. KW and YL: literature search and formal analysis. JL, CX, KW, and YL: writing—original draft preparation. CX and LL: writing—review and editing. All authors read and agreed to the published version of the manuscript.

## References

[B1] Bai Z. Li Y. Yang Y. Xie C. Zhu Z. Xu Y. (2022). The effectiveness of plaza dancing on psychological well-being and ill-being: A systematic review and meta-analysis. *Front. Psychol.* 13:864327. 10.3389/fpsyg.2022.864327 35496162 PMC9051395

[B2] Borquist-Conlon D. S. Maynard B. R. Brendel K. E. Farina A. S. J. (2017). Mindfulness-based interventions for youth with anxiety: A systematic review and meta-analysis. *Res. Soc. Work Pract.* 29 195–205. 10.1177/1049731516684961

[B3] Burke C. A. (2010). Mindfulness-based approaches with children and adolescents: A preliminary review of current research in an emergent field. *J. Child Fam. Stud.* 19 133–144. 10.1007/s10826-009-9282-x

[B4] Carek P. J. Laibstain S. E. Carek S. M. (2011). Exercise for the treatment of depression and anxiety. *Int. J. Psychiatry Med.* 41 15–28. 10.2190/PM.41.1.c 21495519

[B5] Chen X. Zhang B. Jin S.-X. Quan Y.-X. Zhang X.-W. Cui X.-S. (2021). The effects of mindfulness-based interventions on nursing students: A meta-analysis. *Nurse Educ. Today* 98:104718. 10.1016/j.nedt.2020.104718 33454659

[B6] Chen Y. Yang X. Wang L. Zhang X. (2013). A randomized controlled trial of the effects of brief mindfulness meditation on anxiety symptoms and systolic blood pressure in Chinese nursing students. *Nurse Educ. Today* 33 1166–1172. 10.1016/j.nedt.2012.11.014 23260618

[B7] Cheng S. Jia C. Wang Y. (2020). Only children were associated with anxiety and depressive symptoms among college students in China. *Int. J. Environ. Res. Public Health* 17 4035. 10.3390/ijerph17114035 PMC7313008 32517044

[B8] Cheung M. W. L. Ho R. C. M. Lim Y. Mak A. (2012). Conducting a meta-analysis: Basics and good practices. *Int. J. Rheumatic Dis.* 15 129–135. 10.1111/j.1756-185X.2012.01712.x 22462415

[B9] Compen F. Bisseling E. Schellekens M. Donders R. Carlson L. van der Lee M. (2018). Face-to-face and internet-based mindfulness-based cognitive therapy compared with treatment as usual in reducing psychological distress in patients with cancer: A multicenter randomized controlled trial. *J. Clin. Oncol.* 36 2413–2421. 10.1200/JCO.2017.76.5669 29953304

[B10] Crane R. S. Brewer J. Feldman C. Kabat-Zinn J. Santorelli S. Williams J. M. G. (2017). What defines mindfulness-based programs? The warp and the weft. *Psychol. Med.* 47 990–999. 10.1017/S0033291716003317 28031068

[B11] Cui L. He F. Han Z. Yang R. Xiao J. Oei T. P. S. (2016). A brief group cognitive-behavioral program for the prevention of depressive symptoms in Chinese college students. *Int. J. Group Psychother.* 66 291–307. 10.1080/00207284.2015.1111098 38449182

[B12] Dunstan D. A. Scott N. (2020). Norms for Zung’s self-rating anxiety scale. *BMC Psychiatry* 20:90. 10.1186/s12888-019-2427-6 32111187 PMC7048044

[B13] Evans S. Ferrando S. Findler M. Stowell C. Smart C. Haglin D. (2008). Mindfulness-based cognitive therapy for generalized anxiety disorder. *J. Anxiety Disord.* 22 716–721. 10.1016/j.janxdis.2007.07.005 17765453

[B14] Gilbert K. N. (2018). *Meditations on Mindfulness: Cultivating Selves in and Beyond Neoliberalism.* Honor thesis, Wesleyan University, Middletown, CT, 10.14418/wes01.1.70

[B15] Grossman P. Zwahlen D. Halter J. P. Passweg J. R. Steiner C. Kiss A. (2015). A mindfulness-based program for improving quality of life among hematopoietic stem cell transplantation survivors: Feasibility and preliminary findings. *Support. Care Cancer* 23 1105–1112. 10.1007/s00520-014-2452-4 25297466

[B16] Gu Y. Xu G. Zhu Y. (2016). A randomized controlled trial of mindfulness-based cognitive therapy for college students with ADHD. *J. Attent. Disord.* 22 388–399. 10.1177/1087054716686183 28038496

[B17] Guo G. Wang A. (2017). Mindfulness training on college students’ psychological health: Based on Satire Meditation techniques. *J. Neijiang Normal Univ.* 32 7–12.

[B18] Halladay J. E. Dawdy J. L. McNamara I. F. Chen A. J. Vitoroulis I. McInnes N. (2018). Mindfulness for the Mental Health and Well-Being of Post-Secondary Students: A Systematic Review and Meta-Analysis. *Mindfulness* 10 397–414.

[B19] Hayes S. C. Strosahl K. D. Wilson K. G. (1999). *Acceptance and Commitment Therapy: An Experiential Approach to Behavior Change.* New York, NY: Guilford Press.

[B20] Higgins J. P. T. Green S. Chandler J. Cumpston M. Li T. Page M. J. (2019). *Cochrane Handbook for Systematic Reviews of Interventions*, 2nd Edn. New York, NY: Wiley-Blackwell.

[B21] Hofmann S. G. Gómez A. F. (2017). Mindfulness-based interventions for anxiety and depression. *Psychiatr. Clin. North Am.* 40 739–749. 10.1016/j.psc.2017.08.008 29080597 PMC5679245

[B22] Huang J. Lin K. Fan L. Qiao S. Wang Y. (2021). The effects of a self-compassion intervention on future-oriented coping and psychological well-being: A randomized controlled trial in Chinese college students. *Mindfulness* 12 1451–1458. 10.1007/s12671-021-01614-8

[B23] Intarakamhang U. Macaskill A. Prasittichok P. (2020). Mindfulness interventions reduce blood pressure in patients with non-communicable diseases: A systematic review and meta-analysis. *Heliyon* 6:e03834. 10.1016/j.heliyon.2020.e03834 32373739 PMC7191601

[B24] Keng S.-L. Looi P. S. Tan E. L. Y. Yim O.-S. Lai P. S. Chew S. H. (2020). Effects of mindfulness-based stress reduction on psychological symptoms and telomere length: A randomized active-controlled trial. *Behav. Ther.* 51 984–996. 10.1016/j.beth.2020.01.005 33051039

[B25] Khoury B. Lecomte T. Fortin G. Masse M. Therien P. Bouchard V. (2013). Mindfulness-based therapy: A comprehensive meta-analysis. *Clin. Psychol. Rev.* 33 763–771. 10.1016/j.cpr.2013.05.005 23796855

[B26] Kocovski N. L. Fleming J. E. Rector N. A. (2009). Mindfulness and acceptance-based group therapy for social anxiety disorder: An open trial. *Cogn. Behav.Pract.* 16 276–289. 10.1016/j.cbpra.2008.12.004

[B27] Lei F. Chung E. Ling Siew E. (2022). Factors affecting mental health among Chinese college students: A preliminary review of literature. *J. Cogn. Sci. Hum. Dev.* 8 175–185. 10.33736/jcshd.4496.2022

[B28] Li J. Qin X. (2021). Efficacy of mindfulness-based stress reduction on fear of emotions and related cognitive behavioral processes in Chinese University students: A randomized controlled trial. *Psychol. Sch.* 58 2068–2084. 10.1002/pits.22578

[B29] Li M. Ren Y. (2019). Intervention effects of motivation interviewing Chinese modified on the mental health of college students with exercise dependence. *Psychiatr. Q.* 90 447–459. 10.1007/s11126-019-09635-2 31001711

[B30] Lim R. B. C. Zhang M. W. B. Ho R. C. M. (2018). Prevalence of all-cause mortality and suicide among bariatric surgery cohorts: A meta-analysis. *Int. J. Environ. Res. Public Health* 15:1519. 10.3390/ijerph15071519 30021983 PMC6069254

[B31] Liu X. Yi P. Ma L. Liu W. Deng W. Yang X. (2021). Mindfulness-based interventions for social anxiety disorder: A systematic review and meta-analysis. *Psychiatry Res.* 300:113935. 10.1016/j.psychres.2021.113935 33895444

[B32] Liu Y.-C. Li I. L. Hsiao F.-H. (2021). Effectiveness of mindfulness-based intervention on psychotic symptoms for patients with schizophrenia: A meta-analysis of randomized controlled trials. *J. Adv. Nurs.* 77 2565–2580. 10.1111/jan.14750 33450107

[B33] Lo H. H. M. Au A. Cho W. V. Lau E. N. S. Wong J. Y. H. Wong S. Y. S. (2022). Mindfulness-based intervention for caregivers of frail older Chinese adults: A study protocol. *Int. J. Environ. Res. Public Health* 19:5447. 10.3390/ijerph19095447 35564839 PMC9104052

[B34] Lu J. Guan H. Hu X. (2020). Influence of mindfulness decompression therapy on mental health and sleep quality of medical students. *China J. Health Psychol.* 28 1705–1710.

[B35] Marchand W. R. (2012). Mindfulness-based stress reduction, mindfulness-based cognitive therapy, and zen meditation for depression, anxiety, pain, and psychological distress. *J. Psychiatr. Pract.* 18 233–252. 10.1097/01.pra.0000416014.53215.86 22805898

[B36] McCracken L. M. Vowles K. E. (2014). Acceptance and commitment therapy and mindfulness for chronic pain: Model, process, and progress. *Am. Psychol.* 69 178–187. 10.1037/a0035623 24547803

[B37] McShane B. B. Böckenholt U. (2021). Meta-analysis of studies with multiple contrasts and differences in measurement scales. *J. Cons. Psychol.* 32 23–40. 10.1002/jcpy.1236

[B38] Nakagawa S. Lagisz M. Jennions M. D. Koricheva J. Noble D. W. Parker T. H. (2021). Methods for testing publication bias in ecological and evolutionary meta-analyses. *Methods Ecol. Evol.* 13 4–21. 10.1111/2041-210x.13724

[B39] Niu L. (2020). The influence of mindfulness therapy on the interpersonal relationship and empathy ability of college students with mobile phone-dependent. *J. Int. Psychiatry* 47 1161–1165.

[B40] Oei T. P. S. Sawang S. Goh Y. W. Mukhtar F. (2013). Using the Depression Anxiety Stress Scale 21 (DASS-21) across cultures. *Int. J. Psychol.* 48 1018–1029. 10.1080/00207594.2012.755535 23425257

[B41] Page M. J. McKenzie J. E. Bossuyt P. M. Boutron I. Hoffmann T. C. Mulrow C. D. (2021). The PRISMA 2020 statement: An updated guideline for reporting systematic reviews. *Syst. Rev.* 10:89. 10.1186/s13643-021-01626-4 33781348 PMC8008539

[B42] Pan J.-Y. Ye S. Ng P. Y.-N. Lu L. (2020). A randomized controlled trial of cognitive behavioral group prevention program for mainland Chinese university students in Hong Kong. *Res. Soc. Work Pract.* 31 180–193. 10.1177/1049731520962181

[B43] Pang Z. Tu D. Cai Y. (2019). Psychometric properties of the SAS, BAI, and S-AI in Chinese university students. *Front. Psychol.* 10:93. 10.3389/fpsyg.2019.00093 30766501 PMC6365890

[B44] Renner K. H. Hock M. Bergner-Köther R. Laux L. (2018). Differentiating anxiety and depression: the State-Trait Anxiety-Depression Inventory. *Cogn. Emot.* 32, 1409–1423. 10.1080/02699931.2016.1266306 27928931

[B45] Repo S. Elovainio M. Pyörälä E. Iriarte-Lüttjohann M. Tuominen T. Härkönen T. (2022). Comparison of two different mindfulness interventions among health care students in Finland: A randomised controlled trial. *Adv. Health Sci. Educ.* 27 709–734. 10.1007/s10459-022-10116-8 35503145 PMC9063251

[B46] Roemer L. Williston S. K. Eustis E. H. Orsillo S. M. (2013). Mindfulness and acceptance-based behavioral therapies for anxiety disorders. *Curr. Psychiatry Rep.* 15:410. 10.1007/s11920-013-0410-3 24078067

[B47] Shou S. Li Y. Fan G. Zhang Q. Yan Y. Lv T. (2022). The efficacy of cognitive behavioral therapy for tic disorder: A meta-analysis and a literature review. *Front. Psychol.* 13:851250. 10.3389/fpsyg.2022.851250 35401364 PMC8987272

[B48] Smit B. Stavrulaki E. (2021). The efficacy of a mindfulness-based intervention for college students under extremely stressful conditions. *Mindfulness* 12 3086–3100. 10.1007/s12671-021-01772-9 34642590 PMC8498086

[B49] Spitzer R. L. Kroenke K. Williams J. B. W. Löwe B. (2006). A brief measure for assessing generalized anxiety disorder: The GAD-7. *Arch. Int. Med.* 166 1092–1097. 10.1001/archinte.166.10.1092 16717171

[B50] Strauss C. Cavanagh K. Oliver A. Pettman D. (2014). Mindfulness-based interventions for people diagnosed with a current episode of an anxiety or depressive disorder: A meta-analysis of randomised controlled trials. *PLoS One* 9:e96110. 10.1371/journal.pone.0096110 24763812 PMC3999148

[B51] Sturt J. Ali S. Robertson W. Metcalfe D. Grove A. Bourne C. (2012). Neurolinguistic programming: A systematic review of the effects on health outcomes. *Br. J. Gen. Pract.* 62 e757–e764. 10.3399/bjgp12X658287 23211179 PMC3481516

[B52] Sun S. Lin D. Goldberg S. Shen Z. Chen P. Qiao S. (2021). A mindfulness-based mobile health (mHealth) intervention among psychologically distressed university students in quarantine during the COVID-19 pandemic: A randomized controlled trial. *J. Couns. Psychol.* 69 157–171. 10.1037/cou0000568 34264696 PMC8760365

[B53] Vøllestad J. Nielsen M. B. Nielsen G. H. (2012). Mindfulness- and acceptance-based interventions for anxiety disorders: A systematic review and meta-analysis. *Br. J. Clin. Psychol.* 51 239–260. 10.1111/j.2044-8260.2011.02024.x 22803933

[B54] Wan Y. (2020). An analysis of the effect of mindfulness group intervention on college students’ mental health. *Psychol. Mag.* 15 28.

[B55] Wang Y. Jiang C. (2016). Biological mechanisms of mindfulness meditation and physical and mental health. *Chin. Mental Health J.* 30 105–108.

[B56] Yang L. Ren Z. Wang X. Zhang Y. Peng F. Xu J. (2020). Effect of mindfulness-based stress reduction on college students’mental state and sleep during the epidemic of COVID-19. *China J. Health Psychol.* 28 1813–1817.

[B57] Yang X. Liu D. Wang Y. Chen Y. Chen W. Yang C. (2020). Effectiveness of Zhong-Yong thinking based dialectical behavior therapy group skills training versus supportive group therapy for lowering suicidal risks in Chinese young adults: A randomized controlled trial with a 6-month follow-up. *Brain Behav.* 10:01621. 10.1002/brb3.1621 32304353 PMC7303376

[B58] Yang Q. Chi Y. Lu K. Wang B. Wu L. Wang Y. (2018). The study of mindfulness training improving anxiety, self-control ability and learning effect of medical undergraduates in military academy. *Med. J. Air Force* 34 279–280.

[B59] Yi Q.-F. Yan J. Zhang C.-J. Yang G.-L. Huang H. Yang Y. (2022). The experience of anxiety among Chinese undergraduate nursing students in the later period of their internships: Findings from a qualitative study. *BMC Nurs.* 21:70. 10.1186/s12912-022-00847-9 35351129 PMC8961083

[B60] Yu Y. Yan W. Yu J. Xu Y. Wang D. Wang Y. (2022). Prevalence and associated factors of complains on depression, anxiety, and stress in university students: An extensive population-based survey in China. *Front. Psychol.* 13:842378. 10.3389/fpsyg.2022.842378 35418921 PMC8995560

[B61] Zhang T. Fu H. Wan Y. (2014). The application of group forgiveness intervention for courtship-hurt college students: A Chinese perspective. *Int. J. Group Psychother.* 64 298–320. 10.1521/ijgp.2014.64.3.298 24911223

[B62] Zhang X. Zhu H. (2014). The intervention effect of mindfulness-based cognitive therapy on college students with mobile phone addiction. *Stud. Psychol. Behav.* 12 391–394.

[B63] Zhang Z. Jin C. Zhang J. (2021). A meta-analysis of the effects of physical activity intervention on anxiety and depression in Chinese college students. *Psychiatr. Danubina* 33 (Suppl. 6), 395–403.

[B64] Zhu T. Xue J. Montuclard A. Jiang Y. Weng W. Chen S. (2019). Can mindfulness-based training improve positive emotion and cognitive ability in Chinese non-clinical population? A pilot study. *Front. Psychol.* 10:1549. 10.3389/fpsyg.2019.01549 31333552 PMC6619344

[B65] Zoogman S. Goldberg S. B. Hoyt W. T. Miller L. (2015). Mindfulness interventions with youth: A meta-analysis. *Mindfulness* 6 290–302. 10.1007/s12671-013-0260-4

